# A two-component intervention to improve hand hygiene practices and promote alcohol-based hand rub use among people who inject drugs: a mixed-methods evaluation

**DOI:** 10.1186/s12879-021-05895-1

**Published:** 2021-02-25

**Authors:** Salim Mezaache, Laélia Briand-Madrid, Linda Rahni, Julien Poireau, Fiona Branchu, Khafil Moudachirou, Yourine Wendzinski, Patrizia Carrieri, Perrine Roux

**Affiliations:** 1 Aix-Marseille Univ, INSERM, IRD, SESSTIM, Sciences Economiques & Sociales de la Santé & Traitement de l’Information Médicale, Marseille, France; 2 ORS PACA, Observatoire Régional de la Santé Provence-Alpes-Côte d’Azur, Marseille, France; 3 Nouvelle Aube, Marseille, France; 4 Aides, Béziers & Pantin, France; 5 Laboratoire de Recherche Communautaire, Coalition Plus, Pantin, France; 6 ASUD Nîmes, Nîmes, France

**Keywords:** Hand hygiene, Harm reduction, Intervention, Skin and soft tissue infections, People who inject drugs, Alcohol-based hand rubs, Hand sanitizers

## Abstract

**Background:**

Inconsistent hand hygiene puts people who inject drugs (PWID) at high risk of infectious diseases, in particular skin and soft tissue infections. In healthcare settings, handwashing with alcohol-based hand rubs (ABRH) is recommended before aseptic procedures including intravenous injections. We aimed to evaluate the acceptability, safety and preliminary efficacy of an intervention combining ABHR provision and educational training for PWID.

**Methods:**

A mixed-methods design was used including a pre-post quantitative study and a qualitative study. Participants were active PWID recruited in 4 harm reduction programmes of France and followed up for 6 weeks. After baseline assessment, participants received a face-to-face educational intervention. ABHR was then provided throughout the study period. Quantitative data were collected through questionnaires at baseline, and weeks 2 (W2) and 6 (W6) post-intervention. Qualitative data were collected through focus groups with participants who completed the 6-week study.

**Results:**

Among the 59 participants included, 48 (81%) and 43 (73%) attended W2 and W6 visits, respectively. ABHR acceptability was high and adoption rates were 50% (W2) and 61% (W6). Only a minority of participants reported adverse skin reactions (ranging from 2 to 6%). Preliminary efficacy of the intervention was shown through increased hand hygiene frequency (multivariable linear mixed model: coef. W2 = 0.58, *p* = 0.002; coef. W6 = 0.61, *p* = 0.002) and fewer self-reported injecting-related infections (multivariable logistic mixed model: AOR W6 = 0.23, *p* = 0.021). Two focus groups were conducted with 10 participants and showed that young PWID and those living in unstable housing benefited most from the intervention.

**Conclusions:**

ABHR for hand hygiene prior to injection are acceptable to and safe for PWID, particularly those living in unstable housing. The intervention’s educational component was crucial to ensure adoption of safe practices. We also provide preliminary evidence of the intervention’s efficacy through increased hand hygiene frequency and a reduced risk of infection.

**Supplementary Information:**

The online version contains supplementary material available at 10.1186/s12879-021-05895-1.

## Introduction

People who inject drugs (PWID) are at high risk of infectious diseases, including blood-borne viral infections and bacterial or fungal diseases [1–3]. The latter two include skin and soft tissue infections (SSTI), such as cutaneous abscesses, cellulitis and ulcers, which may trigger life-threatening conditions if left untreated (e.g., endocarditis, septicaemia) [4–7]. SSTI are frequent among PWID, with various associated studies estimating abscess prevalence in the previous month between 6 and 32%, and lifetime prevalence up to 68% [8]. SSTI are therefore a leading determinant of healthcare utilization among PWID, with studies highlighting increased hospitalization rates in this population for serious bacterial and fungal infections over the past 10 years [9–11]. Microbiological analyses have shown a predominance of *Staphylococcus aureus* and streptococcus species, followed by anaerobic bacteria and candida species, indicating that most of these pathogens are introduced from the PWID commensal skin and oral flora when introducing the needle inside the skin [2, 5, 11–14]. It is worth noting that PWID also experience disproportionally higher rates of invasive community-associated methicillin-resistant *Staphylococcus aureus* (MRSA) infections [15, 16]. Behavioural studies have confirmed microbiological findings, with associations being found between unhygienic drug-injecting practices and SSTI. More specifically, a lack of skin disinfection at injection sites with alcohol swabs has been extensively associated with increased SSTI risk [17–21]. To a lesser extent, needle licking [17, 18] and inconsistent handwashing prior to injection have also been associated with SSTI [21, 22]. However, other studies failed to find any association between inconsistent hand washing and SSTI [17–19]. This heterogeneity could be explained by the fact that the type of product PWID used to wash their hands was not documented in these studies. Consequently, we do not know whether the products used were actually effective in removing pathogens from hands. Alcohol-based hand rubs (ABHR), also known as hand sanitizers, are considered the gold-standard by the World Health Organization (WHO) for hand hygiene of healthcare workers [23]. ABHR showed in vivo superiority in reducing hand contamination when compared with plain soaps and antimicrobial detergents, including chlorhexidine, povidone-iodine and triclosan [24–27]. The advantages of ABHR for hand hygiene include fast-acting disinfection, quick use, optimal antimicrobial spectrum and no need for water [28]. Since their development in the 1990s, ABHR have been extensively evaluated in healthcare settings, and results show that hand hygiene with ABHR among healthcare workers is effective in reducing healthcare-associated infection rates, including MRSA infections [29–31]. It is noteworthy that ABHR are not restricted to healthcare settings, as campaigns have also been implemented in community settings, mainly for the purpose of gastrointestinal or respiratory infection control, including the recent COVID-19 pandemic [32, 33]. The WHO guidelines outline “5 moments” when ABHR use is required. The second moment, entitled “before a clean/aseptic procedure”, matches the context of PWID intravenous injecting practices, suggesting that ABHR use should be recommended before each injection in order to limit risks for PWID [34]. In addition to its antimicrobial efficacy, ABHR has the potential to overcome two known barriers to practicing risk reduction, namely lack of access to water and the limited time allowed to hand hygiene before injection [35]. To date, no study has evaluated interventions specifically promoting ABHR use for hand hygiene among PWID. This is why we aimed to design, implement, and evaluate an intervention, based on healthcare safety standards, to improve hand hygiene practices in PWID and promote ABHR use prior to drug injection.

## Methods

### Aims and study design

**Fig. 1:**
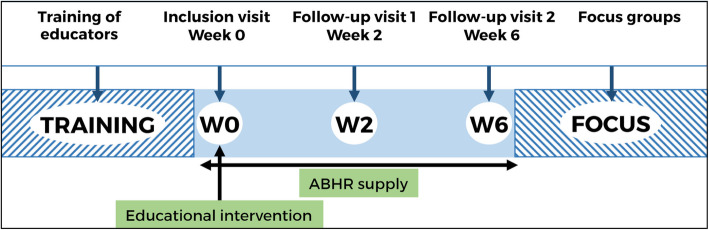
Design of the study

We used a convergent parallel mixed-methods study design to evaluate the acceptability, safety and preliminary efficacy of an intervention combining ABHR provision in mono-dose containers (hereafter called “*MONO-RUB*”) with brief educational sessions in order to promote good hand hygiene practices of PWID. This particular mixed-methods design consists in concomitant quantitative and qualitative data collection, separate analyses and triangulation [36]. This approach was chosen to ensure the broadest possible understanding of participants’ beliefs and perceptions about the intervention. The quantitative component, which constituted the main part of the study, consisted of a pre-post intervention, multicentre, non-comparative study (i.e.*,* no control group was recruited). The study design is depicted in Fig. 1. Each participant was followed up for 6 weeks and had 3 study visits (i.e.*,* baseline, 2 weeks (W2) and 6 weeks (W6) after baseline). The qualitative component was based on focus group discussions with participants who had completed the quantitative study.

### Settings and participants

The study took place in 4 harm reduction (HR) programmes in four cities in southern France (i.e.*,* Arles, Béziers, Marseille and Nîmes) between February 2019 and January 2020. These programmes were selected to ensure diversity of participant profiles. They are run by community-based organizations providing low-threshold HR services with onsite or outreach activities. Routine activities of these programmes include first-line PWID support through the provision of injecting equipment, advice about safer drug use, HIV and HCV rapid testing, primary healthcare and assistance for social entitlements. Before this study, these programmes did not provide any equipment or counselling specifically intended to improve hand hygiene practices.

Participants were conveniently recruited from the active client base of each HR programme. First, individuals who indicated interest were screened for eligibility by programme staff. Eligibility criteria were as follows: aged 18 years or over, active PWID (defined as having injected drugs during the previous week) and willing to participate after being informed about the study. Exclusion criteria included being allergic to alcohol and staying only a short while in the city (< 6 weeks). With regard to the focus group discussions, we included voluntary participants who completed the 3 quantitative study visits. Participants were compensated €10 for each questionnaire completed and for attending the focus groups.

### Intervention

The two-component intervention was co-designed with the help of both hand hygiene experts and members of the PWID community to ensure consistency with up-to-date scientific evidence and to match PWID needs. Overall, the intervention covered three elements of the behaviour change wheel: (i) *enablement*, through increased availability of ABHR; (ii) *education*, through increased knowledge and understanding about hand hygiene and (iii) *training*, through the teaching of appropriate hand-rubbing techniques [37].

The first component was the provision of *MONO-RUB*. The active ingredient was ethanol (70% w/w) and the formulation included moisturizing and emollient agents to limit skin irritation. The solution was contained in an innovative single-use 3.5 mL sachet with an easy-to-use one-hand opening system (Fig. 1). Participants were provided *MONO-RUB* as much as necessary during the study period. At first visit, they were provided sufficient stock of *MONO-RUB* to cover all injections until the next planned encounter with the HR programme.

**Fig. 2:**
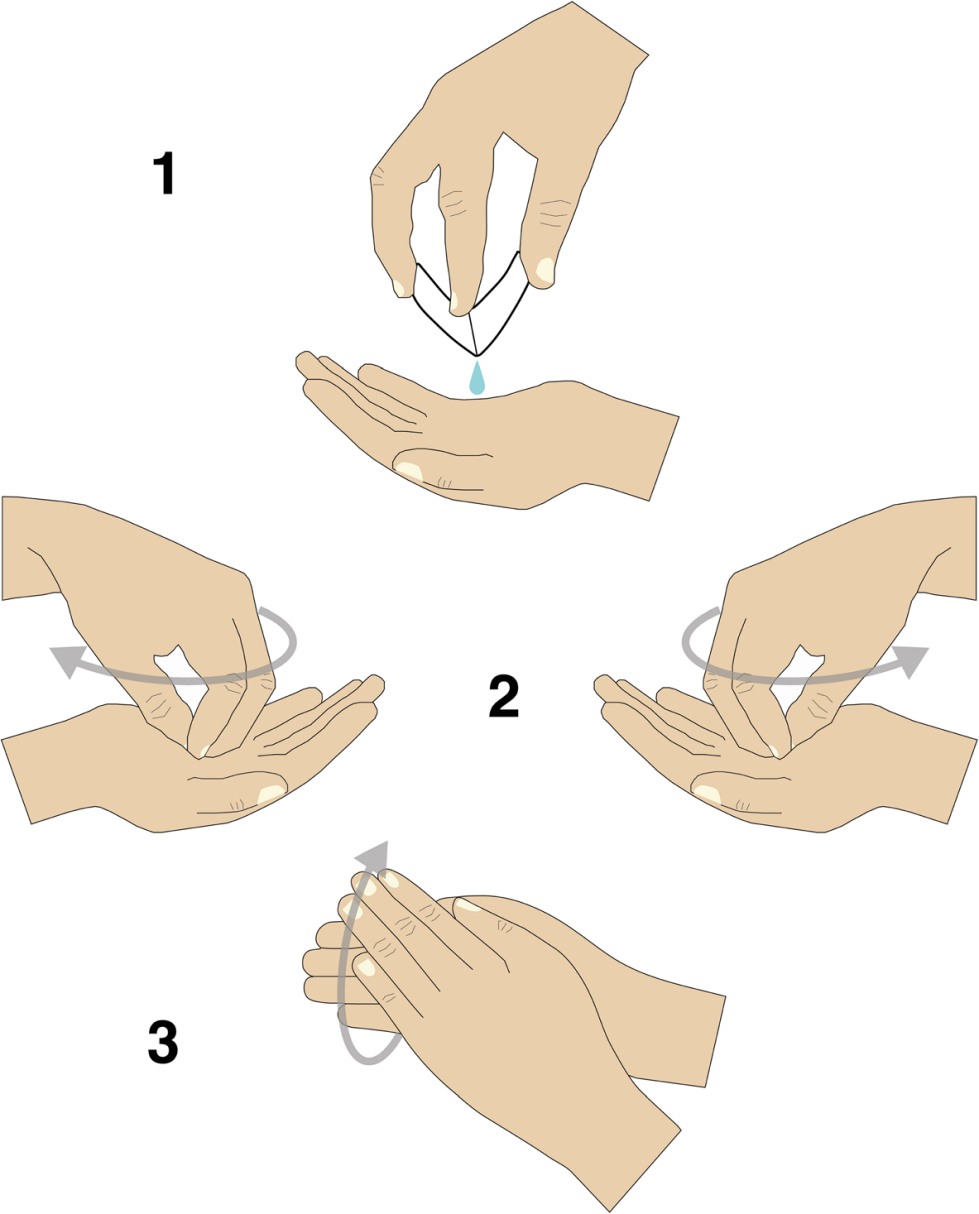
The simplified 3-step hand hygiene technique using *MONO-RUB*. (1) Pouring the product from one hand into the palm of the other hand; (2) Using fingertips of each hand to rub product into the palm of the other hand; (3) Rubbing both hands together for at least 15s

Providing ABHR without implementing a concomitant behavioural change strategy is known to be suboptimal [38]. Accordingly, we integrated educational sessions of hand hygiene as a second intervention component. This consisted of brief individual face-to-face sessions with trained educators taking place in the HR centres or through outreach. Educators included trained social workers, nurses and peer-educators. Between 2 and 4 educators were trained in each HR programme (12 in total). It consisted of a 3 h long face-to-face training delivered by a research pharmacist (SM). They all received a booklet detailing all steps of the intervention. After participant inclusion and baseline face-to-face questionnaires, educators conducted one-to-one educational sessions intended to increase participant knowledge about the risks of poor hand hygiene before injection, the advantages of using ABHR, and how to use it correctly. Participants were also taught a simplified 3-step hand-rubbing technique, as studies have shown that the complexity of the WHO-recommended 6-step technique for healthcare settings often leads to poor adherence by healthcare workers [39]. Furthermore, recent studies have shown the efficacy of a simplified 3-step technique on both microbiological and hand hygiene frequency endpoints [40–42]. First, product is poured into the palm of one hand and rub the fingertips of the opposite hand. Solution surplus is then poured into the palm of the other hand, and fingertips of the opposite hand are rubbed. The full hands then rub each other completely for at least 15 s (Fig. 2). At the end of the educational session, participants were provided with a flyer depicting the 3-step technique.

### Outcomes

The primary study outcome was a composite of the different measurements of the intervention’s acceptability. It was assessed by self-reported measures combined with observed behaviours at the two follow-up visits (W2 and W6). First, Likert scales (0–10) were used to assess satisfaction, usability, perceived usefulness, willingness to show other people and willingness to use in future. Second, we examined rates of self-reported adoption of *MONO-RUB* as the main product for hand hygiene. Finally, adherence to the 3-step hand-rubbing technique was measured through direct observation at W2 and W6.

Secondary outcomes included intervention safety and preliminary efficacy. Safety measurements included skin tolerability, rates of adverse events and diversion, and were measured at the two follow-up visits. Efficacy was evaluated through pre- and post-intervention comparison of self-reported hand hygiene frequency prior to injection and rates of injecting-related complications.

### Data collected

Quantitative data were collected by trained interviewers through face-to-face administered questionnaires at baseline (i.e.*,* prior to the intervention), first follow-up visit (W2) and second follow-up visit (W6). These questionnaires were specifically developed for this study and available in supplementary information. Data on sociodemographic and economic characteristics and current drug use patterns were only collected at baseline. The main product used by participants to perform hand hygiene and the frequency of hand hygiene (measured on a 5-point Likert scale ranging from 0 = *Never* to 4 = *Always*) during the two previous weeks were recorded at all three visits. Adherence to the 3-step hand-rubbing technique was recorded through direct observation of participants by the interviewer at W2 and W6. Experiencing injecting-related complications (i.e.*,* SSTI, cotton fever, sepsis or endocarditis) during the previous month was recorded at baseline and W6. Adverse skin reactions (e.g.*,* redness, dryness, allergies) and other adverse events related to the use of *MONO-RUB* were self-reported at W2 and W6. Diversion of *MONO-RUB* (e.g.*,* used to clean another body part, used as a combustible, deliberately ingested) was collected at W2 and W6.

Qualitative data were collected through two focus groups with 10 participants (5 for each focus group) who had completed both quantitative follow-up study visits. Of these 10 participants, 9 were males and age was ranged between 25 to 51 years old. These focus group took place in two different programmes and lasted for about 1 h each. An interview guide was used with open-ended questions exploring participant acceptability (e.g.*, what are the (dis) advantages of this hand hygiene method?*), changes in practices after the intervention (e.g.*, to what extent this intervention changed your practice?*), knowledge gained and the intervention’s relevance to personal needs.

### Analysis

Quantitative data analyses first included a description of participants’ baseline characteristics and comparison between those who completed follow-up and those who did not. Likert scales were described in terms of mean and standard deviation. Adoption potential was evaluated by assessing whether the proportion of participants who reported using *MONO-RUB* as the main method for hand hygiene at W2 and W6 was greater than 20% at *p* <  0.05 using a single-tailed McNemar test for paired data. Adoption rates between 10 and 20% are generally required for the diffusion of innovations [43]. Pre-post hand hygiene frequency change was modelled using a multivariable linear mixed model with a random intercept per subject for all available observations. This type of model is best suited for pre-post intervention studies, as it takes into account correlation between repeated measures within subjects and loss to follow-up, and therefore ensures adherence to the intention-to-treat principle [44]. The main explanatory variable was the follow-up variable with the baseline visit as a reference. The model was a priori adjusted for potential confounders including age, gender, educational level, housing stability and injection frequency. In addition, two predefined subgroup analyses were performed to investigate differential efficacy according to age (< 30 vs. ≥ 30 years) and housing (stable vs. unstable). We selected these two variables because younger PWID and those living in unstable conditions are often more affected by injecting-related harms [45, 46]. Interactions between subgroup variables and follow-up were tested for in the mixed linear model. Finally, the effect of the intervention on injecting-related complications was assessed using a multivariable logistic mixed model. Explanatory and confounder variables were the same as those described above. Sensitivity analysis with complete cases (i.e.*,* participants not lost to follow-up) was performed on the two efficacy measurements and yielded similar results (data not shown).

Qualitative data from the focus groups were audio-taped with participants’ oral informed consent, then transcribed verbatim and anonymized. Thematic analysis was performed. Transcripts were read and coded by the two investigators (SM and PR) inductively in order to identify emerging themes. These themes were then categorized as barriers or facilitators to acceptability or efficacy of the intervention as higher-level themes. The research investigators identified key illustrating verbatim quotations. After separate analyses, quantitative and qualitative data were integrated by side-by-side comparison. Research team discussed individual findings of quantitative and qualitative data and then performed triangulation to identify convergence or divergence. In the Discussion section, we outline whether data converged or diverged between the two analyses.

## Results

### Quantitative study

#### Study population and visits

The 59 eligible PWID were all included in the study. Table 1 shows baseline participants’ characteristics. Median age was 41 years (interquartile range (IQR): 35–47), the majority were males (76%), unemployed (83%), and had an educational level below high-school diploma (73%). Almost half (46%) had unstable housing and 25% had slept in the street during the previous month. A majority (76%) were daily drug injectors and the main injected product was cocaine (73%), followed by morphine sulfate (41%) and buprenorphine (39%). With respect to lifetime drug-related complications, 55% had a history of HCV infection, 71% SSTI, 76% cotton fever and 10% endocarditis or sepsis.

**Table 1:** Baseline characteristics of included participants (*n* = 59) and comparison between participants with at least one follow-up assessment (*n* = 48) and those lost to follow-up (*n* = 11)

Characteristics	At least one follow-up assessment	Lost to follow-up	***P***	Total
	n (%) or median [IQR]	n (%) or median [IQR]		n (%) or median [IQR]
**Gender (Male)**	37 (77%)	8 (73%)	0.76	45 (76%)
**Age (years)**	43.5 [35–47.5]	38 [33–45]	0.17	41 [35–47]
**Education (< High School Diploma)**	32 (67%)	11 (100%)	0.03	43 (73%)
**Unstable housing (Yes)**	16 (33%)	11 (100%)	< 0.001	27 (46%)
**Having slept in the street**^**a**^ **(Yes)**	10 (21%)	5 (45%)	0.09	12 (25%)
**Employment (Yes)**	9 (19%)	1 (9%)	0.67	10 (17%)
**Injection in public places (Yes)**	16 (13%)	4 (36%)	0.08	10 (17%)
**Daily drug injection**^**a**^ **(Yes)**	36 (77%)	8 (73%)	0.30	44 (75%)
**Number of daily injections**^**a**^	4 [2–5]	3 [2–4]	0.59	4 [2–5]
**Drugs injected**^**a**^				
Heroin	9 (19%)	3 (73%)	0.53	12 (20%)
Buprenorphine	20 (42%)	3 (27%)	0.38	23 (39%)
Morphine sulfate	19 (40%)	5 (45%)	0.72	24 (41%)
Cocaine	34 (71%)	9 (82%)	0.46	43 (73%)
Methylphenidate	3 (6%)	2 (18%)	0.23	5 (9%)
**Current opioid agonist treatment (Yes)**	35 (73%)	9 (82%)	0.71	44 (76%)
**History of HCV infection**			0.94	
No	21 (45%)	5 (45%)		26 (45%)
Yes, cured	15 (32%)	3 (27.5%)		18 (31%)
Yes, current	11 (23%)	3 (27.5%)		14 (24%)
**Injecting-related complications (lifetime)**				
Abscesses and other SSTI	32 (67%)	10 (91%)	0.15	42 (71%)
Cotton fever	36 (75%)	9 (82%)	0.63	45 (76%)
Endocarditis or sepsis	5 (10%)	1 (9%)	0.90	6 (10%)

*Abbreviations*: *IQR* Interquartile range, *HCV* Hepatitis C Virus, *SSTI* Skin and soft tissue infections

^a^Previous month

In terms of attrition rate, 11 (19%) were lost to follow-up before W2 and 5 (8%) between W2 and W6, respectively. We were able to collect reasons for dropout for 4 of them: 2 were admitted to long-term detoxification, 1 was incarcerated and 1 moved to another city. We are not aware of any dropout due to adverse events. We compared participants with no follow-up assessment (i.e., lost to follow-up at W2) with those having at least one (Table 1). The former were more likely than the rest of the participants to report a low educational level (i.e.*,* < high-school diploma) and unstable housing. It is worth noting that hand hygiene frequency and injecting-related complications at baseline did not differ significantly (*p* > 0.05) between the two groups.

#### Acceptability

Participant responses to Likert scales are presented in Table 2. General satisfaction was high at the two follow-up visits (mean score 8.8 and 9.0 at W2 and W6, respectively). Similar results were found for other measures with mean scores ranging between 8.3 and 9.3. The product most used for hand hygiene at baseline was plain soap (47%) and 14% of participants reported never performing hand hygiene (Table 3). Post-intervention, 50 and 61% of participants reported adopting *MONO-RUB* as the main product for hand hygiene at W2 and W6, respectively. These proportions were significantly higher than our predetermined 20% threshold for assessing adoption potential (*p* < 0.01). It is worth noting that some participants continued to use alcohol or chlorhexidine wipes. Finally, with regard to the 3-step hand-rubbing technique, at W2 71% reported having performed the fingertips step, this value increasing to 88% at W6. The other 2 steps were properly performed by the vast majority of participants (Table 2).

**Table 2:** Post-intervention acceptability and safety outcomes

	Week 2 (***n*** = 48)	Week 6 (***n*** = 43)
	**Mean (SD)**	**Mean (SD)**
**On a scale from 0 (not at all) to 10 (absolutely)**		
Overall, are you satisfied with the hand washing method? (packaging, product and technique)	8,8 (1,7)	9,0 (1,4)
Do you think this method was better than the one you were used to?	8,6 (1,8)	8,8 (1,7)
Do you think the packaging was easy to use?	9,3 (1,4)	9,3 (1,1)
Do you think the 3-step technique was easy?	8,4 (2,1)	8,6 (1,6)
Do you think you would show other people this method?	8,3 (2,1)	8,5 (1,8)
Do you think you would continue to use this method if it were available free of charge?	9,3 (1,5)	9,2 (1,5)
**Adherence to the hand-rubbing technique**^**a**^	**n (%)**	**n (%)**
Step 1: All the product poured into the hand (3.5 ml)	44 (92%)	40 (93%)
Step 2: Rubbing of fingertips	34 (71%)	38 (88%)
Step 3: Rubbing of the rest of the hands	45 (96%)	40 (93%)
Rubbing for at least 15 s	39 (83%)	41 (95%)
**Safety: Adverse skin reactions**	**n (%)**	**n (%)**
Dryness	2 (4%)	2 (5%)
Redness or burning feeling	2 (4%)	2 (5%)
Itching	3 (6%)	1 (2%)
**Safety: Diversion of product**	**n (%)**	**n (%)**
Personal hygiene	12 (25%)	8 (19%)
Injection site cleaning	8 (17%)	6 (14%)
Used as combustible	2 (4%)	2 (5%)

Nb: all measurements refer to the 2 weeks before the visit

*Abbreviation*: *SD* Standard deviation

^a^Measured through direct observation

**Table 3:** Product most used and hand hygiene frequency before injection, pre- (i.e., baseline) and post- (i.e., W2 and W6) intervention measurements

	Baseline (***n*** = 59)	Week 2 (***n*** = 48)		Week 6 (***n*** = 43)	
	n (%)	n (%)	Δ^**a**^ (%)	n (%)	Δ^**a**^ (%)
**Product most used for hand hygiene**					
No hand hygiene	8 (14%)	0	*−14%*	0	*−14%*
Only water	8 (14%)	4 (9%)	*−5%*	2 (5%)	*−9%*
Water and plain soap	27 (47%)	14 (30%)	*−17%*	9 (22%)	*−25%*
ABHR (other than *MONO-RUB*)	6 (11%)	0	*−11%*	0	*−11%*
Alcohol and/or Chlorhexidine wipes	8 (14%)	4 (9%)	*−5%*	5 (12%)	*−2%*
*MONO-RUB*	^b^	24 (50%)	*+ 50%*	25 (61%)	*+ 61%*
**Frequency of hand hygiene before injection**					
Never	8 (14%)	0	*−14%*	0	*−14%*
Less than half of the time	12 (21%)	6 (12.5%)	*−8.5%*	8 (19%)	*+ 6.5%*
Half of the time	11 (19%)	6 (12.5%)	*−6.5%*	1 (2%)	*−10.5%*
More than half of the time	5 (8%)	14 (29%)	*+ 21%*	13 (31%)	*+ 23%*
Always	22 (38%)	22 (46%)	*+ 8%*	20 (48%)	*+ 10%*

Missing data ranged from 2 to 5%

^a^Compared with baseline (i.e., pre-intervention)

^b^Absent at baseline

#### Safety

Only a minority of participants reported adverse skin reactions (Table 2). No serious adverse event was reported during follow-up. Diversion of *MONO-RUB* was infrequent the most common reasons being personal hygiene and injection-site cleaning. No ingestion of the product was reported.

#### Preliminary efficacy

**Fig. 3:**
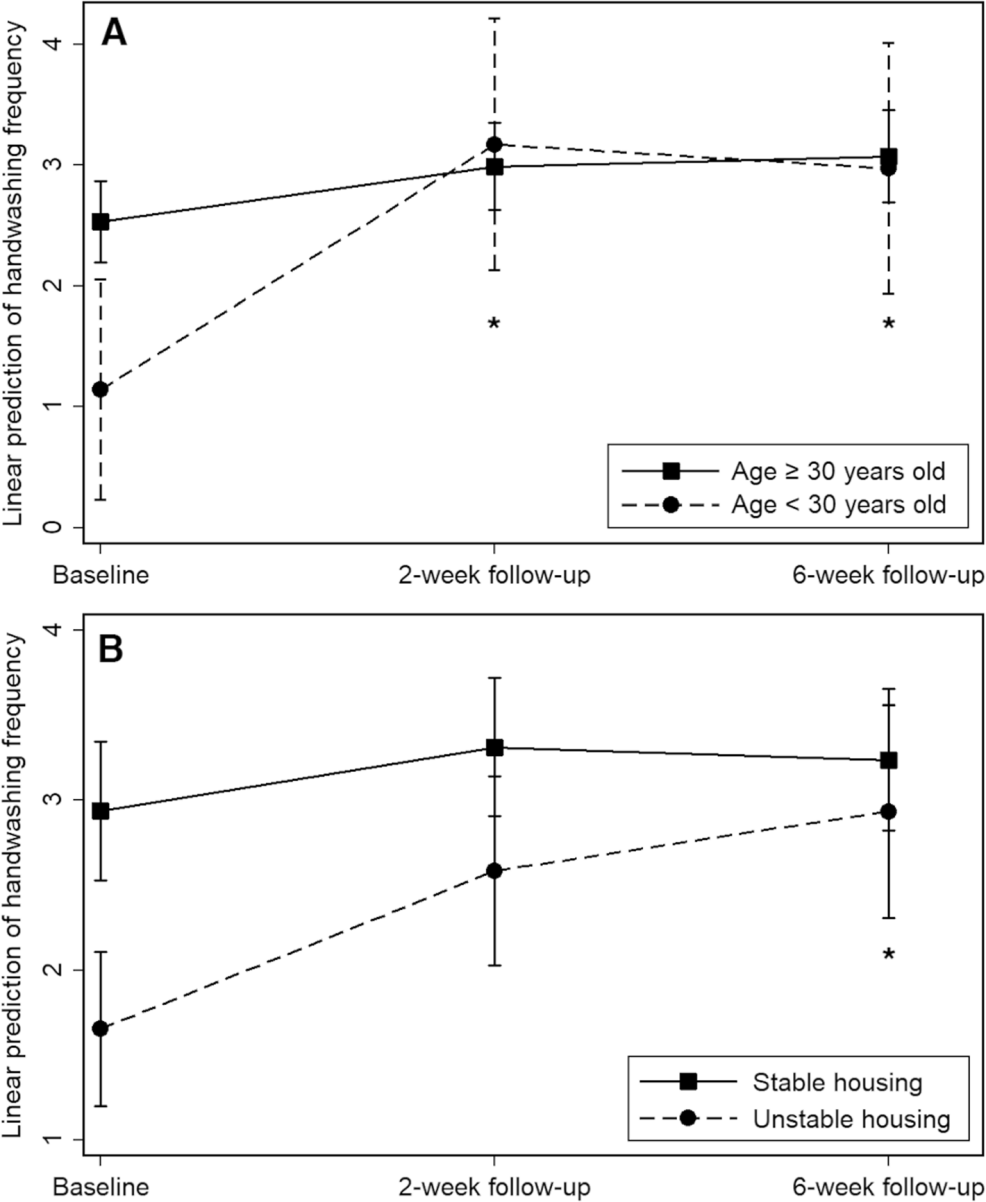
Predictive margins of mixed linear models with interaction between age (**a**) and follow-up, and housing (**b**) and W6 follow-up visit

Our first preliminary efficacy measurement was the pre-post intervention change in frequency of hand hygiene before injection (Table 3). At baseline, 14% of participants reported never performing hand hygiene prior to injection, while 46% reported performing it “always” or “most of the time”. The latter number rose to 75 and 79% at W2 and W6, respectively. In the multivariable linear mixed model, which we adjusted for potential confounders, hand hygiene frequency was significantly higher at W2 (coef. = 0.58, 95%CI: 0.22–0.95, *p* = 0.002) and W6 (coef. = 0.61, 95%CI: 0.23–0.99, *p* = 0.002) than at baseline (Table 4). Moderator analyses showed a significant interaction effect between age and follow-up (interaction coef. W2 (ref. age > 30) = 1.57, *p* < 0.01; interaction coef. W6 (ref. age > 30) = 1.28, *p* < 0.05) indicating a greater increase in hand hygiene frequency for participants aged under 30 years old than for older participants (Fig. 3). Similarly, a significant interaction effect was observed between housing and the second follow-up visit at W6, revealing a greater increase in hand hygiene frequency for participants reporting unstable housing (interaction coef. W6 (ref. stable housing): 0.98, *p* < 0.05; not significant at W2).

**Table 4:** Multivariable models of efficacy outcome, linear mixed model (measurement 1), logistic mixed model (measurement 2), fixed effects

	Multivariable models^**a**^		
	**Coef.**	**(95%CI)**	***p***
**Measurement 1: Hand hygiene frequency before injection**^**b,c**^			
Intercept	3.06	(1.44–4.68)	
Baseline visit (pre-intervention)	0		
2-week follow-up visit (post-intervention)	0.58	(0.22–0.95)	0.002
6-week follow-up visit (post-intervention)	0.61	(0.23–0.99)	0.002
**Measurement 2: Rates of injecting-related infection**^**d**^	**aOR**	**(95%CI)**	***p***
Baseline visit (pre-intervention)	1		
6-week follow-up visit (post-intervention)	0.23	(0.07–0.80)	0.021

Measurement 1, *n* = 58 participants, *n* = 148 visits; Measurement 2, *n* = 59 participants, *n* = 102 visits)

*Abbreviation*: *aOR* Adjusted Odds Ratio

^a^All models were a priori adjusted for age, gender, education level, housing stability and injection frequency

^b^Previous 2 weeks

^c^5-point Likert scale ranging from 0 = *Never* to 4 = *Always*

^d^(during the previous month)

The second efficacy measurement was rates of injecting-related infections (i.e.*,* SSTI, cotton fever, endocarditis or sepsis). At baseline, 56% of participants reported at least one such complication during the previous month. This number dropped to 33% after 6 weeks of follow-up. In the multivariable logistic mixed model adjusted for potential confounders, participants were significantly less likely to report injecting-related complications at W6 compared with baseline (adjusted Odds Ratio (aOR) = 0.20, 95%CI: 0.07–0.80, *p* = 0.021).

### Qualitative study

The qualitative study consisted of two focus group discussions with a total of 10 participants who completed all 3 visits (i.e.*,* baseline, W2 and W6) of the quantitative study. It aimed to complement the quantitative study by further exploring participants’ acceptability of the intervention. Focus groups identified 5 themes as drivers or barriers of acceptability of *MONO-RUB.* First, *usability* refers to factors related to *MONO-RUB* and influencing product’s ease of use. *Usefulness* theme gathers information about the extent *MONO-RUB* is matching people needs. *Safety and skin reaction* refers to skin tolerability of the product and potential diversion of use. *Psychological and cognitive factors* includes individual factors that influence intervention’s acceptability such as perceived efficacy or habits. Finally, *socio-environmental influences* refers to the physical and social environment, including context of drug use, influencing intervention’s individual outcomes (Table 5).

**Table 5:** Key themes and illustrative quotes

Themes	***Facilitators***	***Barriers***
***Usability***	*“it is handy and easy to carry in a trouser pocket or a backpack”*	*“there’s too much inside, you don’t use all the liquid. Sometimes, it flows out on the side”*
	*“the opening system is ingenious making the product easy to use and easy to open”*	*“it would be good to make it less liquid*”
***Usefulness***	*“you don’t need water, you don’t need a sink, wherever you are you can use it”*	*“unlike the product, with the wipes, you can clean your hands when they’re dirty”*
***Safety and skin reaction***	*“the product is good, it dries fast, it’s not greasy; I like the smell and that it leaves the hands soft”*	*“I know someone who used it for heating the drug”*
***Psychological and cognitive factors***	*“after the use, I had the feeling that the hands were really clean”*	*“I do it more as if I’m washing my hands, without necessarily insisting on fingertips”*
	*“if you have it nearby, it’s easier to remember to use it”*	*“the wipes are better to wash when my hands are dirty”*
		*“it is hard to change habits”*
***Socio-environmental influences***	*“I was proud to have it so I show it to other people”*	“*if you’re on a coke session, every 10 min you’ve your head in the bag, you’re not going to be washing your hands every 10 min”*

#### Facilitators

In participant discourses, good usability emerged as the main facilitator of the intervention’s acceptability. Participants saw the product as “*handy and easy to carry”* as it could be carried “*in a trouser pocket or a backpack*”. The packaging was seen as “*easy to use and easy to open”* which also contributed to its acceptability. The usefulness of *MONO-RUB* in case of a lack of water was also mentioned: “*You don’t need water, you don’t need a sink, wherever you are you can use it”*. No participant reported adverse skin reactions. On the contrary, they were very positive about skin sensations after applying *MONO-RUB*: “*the product is good, it dries fast, it’s not greasy; I like the smell and that it leaves the hands soft*”. Moreover, several participants could “*feel the efficacy”* and had “*the feeling that the hands were really clean*” after using *MONO-RUB*. Finally, peer dissemination was reported as some participants had *“shown other people*” the product but “*without necessarily showing the technique for washing*”.

#### Barriers

Barriers to the intervention’s acceptability were: Participants reported concerns about the volume of solution in the packaging: “*there’s too much inside, you don’t use all the liquid. Sometimes, it flows out on the side*”. One participant commented on the consistency, suggesting “*to make it less liquid*”. The fingertips step of the technique was inconsistently performed, one respondent stating “*do it more as if I’m washing my hands, without necessarily insisting on fingertips*”. Being in a hurry to get a hit prevented participants from performing hand hygiene properly: “*when you’re in a hurry and you’re hooked, you just don’t think about washing our hands*”. This seems to be related to the consumption of stimulants, which was associated with very frequent injecting as illustrated by one participant: “*if you’re on a coke session, every ten minutes you’ve your head in the bag, you’re not going to be washing your hands every ten minutes. You’ll do it once at the beginning of the session and maybe a second time*”. The perceived inefficacy to wash visibly dirty hands with *MONO-RUB* was also a concern. Participants reported that alcohol and chlorhexidine wipes were much more effective at doing this: *“with the wipes, you can clean your hands when they’re dirty”*. Finally, persistent habits and routine could be barriers to change as it might take time for “*every new step in the cooking process”* - to cite the words of one participant - to be accepted. Another respondent suggested that older users were more reluctant to change than younger ones: “*it’s good for the new generations who are going to adopt this practice. It’s more complicated for older generations*”.

## Discussion

This study aimed to assess the acceptability, safety and preliminary efficacy of an intervention promoting the use of ABHR for hand hygiene prior to injection among PWID. Results showed high acceptability, reflected in more than half the participants adopting *MONO-RUB* as their main hand hygiene product, and good adherence to the 3-step rubbing technique. Safety was also satisfactory, with very low rates of adverse skin reactions and no serious adverse event reported. Finally, the intervention seemed to be effective in improving hand hygiene frequency and reducing the risk of injecting-related complications. As the study was not exactly designed to assess the efficacy of the intervention, these latter results must be interpreted with caution.

Acceptability was high for all our outcome measurements and we identified key factors influencing it. First, participant satisfaction with *MONO-RUB* and the fact that it met what was a previously unmet need in this population, especially when water was not available, were both linked to acceptability. The fact that the intervention was co-designed with field workers and PWID peers may have contributed to its success in meeting PWID needs. We support previous suggestions advocating the inclusion of peers in the development and evaluation of HR interventions [47]. No specific hand hygiene product is currently present in injecting kits accessible in France. A next step would be these single-dose sachets to be added to kits to ensure they are easily available. Second, good usability of the sachets also appeared as a main facilitator of acceptability in both quantitative and qualitative analyses. Research in ergonomics has highlighted several factors which minimize cognitive and physical efforts and influence appropriate use of health devices, including usability [48]. For instance, poor usability has been associated with low adherence to the use of wearable ABHR dispensers among healthcare workers [49]. Similarly, PWID in another study were reluctant to use membrane filters instead of cotton balls partly because of their poor usability [50]. Third, a majority of participants properly performed the simplified 3-step hand-rubbing technique at follow-up visits, reflecting its acceptability to them. Nevertheless, fewer participants performed the second step (i.e.*,* rubbing fingertips of one hand on the palm of the other) correctly, despite it being crucial for antimicrobial efficacy. This was confirmed by qualitative findings. It would therefore appear essential to place greater emphasis on this particular step during educational sessions in future. The rate of *MONO-RUB* adoption in our study was high but somewhat limited by the product’s perceived inefficiency on visibly dirty hands. Indeed, some participants were likely to report the use of alcohol or chlorhexidine wipes to clean hands in this state. A recent study showed the inferiority of wiping to hand rubbing in terms of reducing bacterial count [51]. In the case of visibly dirty hands, the WHO recommends that people first wash their hands with plain soap and water and then use ABHR [23]. However, recent studies have shown similar efficacy for ABHR and plain soap on dirty hands, while further research is required to assess ABHR efficacy on dirty hands [52, 53]. Our study also showed the potential for widespread diffusion of the intervention through participants’ social networks. Reports have highlighted that PWID themselves can spread HR knowledge and interventions to their peers [54, 55]. However, in our qualitative findings, some respondents who had shown other people the ABHR did not teach them the proper 3-step technique, which underlines the central role of the educational sessions. Finally, acceptability of hand hygiene products is influenced by adverse skin reactions - such as dryness and irritation - which are often cited as a barrier to hand hygiene among healthcare workers [56]. Our results showed very low rates of adverse skin reactions over the 6 weeks of follow-up, something which could have greatly influenced the intervention’s acceptability.

Our findings provide preliminary evidence for the intervention’s efficacy. First, hand hygiene frequency prior to injection significantly increased. In addition, both quantitative and qualitative analyses identified housing, and to a lesser extent, age, as moderators of efficacy. Young PWID, defined in our study as those under 30 years old, were more likely to increase hand hygiene frequency than older PWID. Similar results were reported in a study evaluating acceptability of low dead space syringes where older users were more reluctant to change practices [57]. Habits and rituals more embedded among older and long-term PWID could explain these observations. In the future dissemination of the intervention, specific focus should be placed on these users. Unstable housing was also associated with greater intervention efficacy in terms of hand hygiene frequency. This was to be expected since people living in unstable conditions might have less access to hygiene facilities and therefore would have used *MONO-RUB* more frequently [58]. There is extensive evidence that environmental factors are related to unsafe practices and injecting-related diseases [59, 60]. This intervention therefore has potential to reduce this risk among PWID living in unstable conditions. These results should be interpreted with caution since there was large baseline difference between subgroups. Finally, the intervention also significantly reduced the rates of injecting-related complications. This result should be interpreted with caution since: (i) no medical examination was performed; (ii) no control group was included; (iii) a part of the complications recorded at W6 might not have been incident. More research is needed to confirm all the above-mentioned preliminary insights. The improved hand hygiene practices with ABHR which this intervention offers also have the potential to reduce community transmission of gastrointestinal and respiratory infections, including influenza and coronavirus infections, in this vulnerable population.

Our results should be interpreted in light of the following limitations. First, the non-comparative design of the study limits formal causal inferences, and consequently observed changes cannot be fully attributed to the intervention. Nevertheless, given the short follow-up we can assume that the only factor influencing hand hygiene practices was the intervention. Having said that, we cannot rule out a placebo effect, which has been observed in previous comparative studies [61, 62]. Accordingly, adoption rates and efficacy might be overestimated. Second, findings were mainly based on self-reported data, which may have introduced social-desirability and recall biases. However, numerous studies have shown the reliability of self-reported data among PWID, even for medical data such as SSTI occurrence [63, 64]. Finally, although the short follow-up period may have been a strength for causal inferences, it prevented evaluation of the outcomes over the long term. More research is needed to evaluate the sustainability of the changes induced by this intervention.

## Conclusions

This intervention, which combines the provision of ABHR with brief educational training, was acceptable and safe among a population of active PWID in France. Our results also suggest the intervention’s efficacy in increasing hand hygiene frequency and reducing injection-related complications, although further research is needed to confirm these results. This study also pointed out that HR interventions targeting hand hygiene are lacking, and that the simple intervention described here has the potential to address currently unmet needs, particularly for people living in unstable conditions. Finally, we showed that healthcare standards can be transferred to the community context, but only with peer-led adjustments.

## Supplementary Information

**Additional file 1: Supplementary Information.** Questionnaires used during the study.

## Data Availability

Please contact corresponding authors for requests of datasets or material.

## Abbreviations

ABHR: Alcohol-based hand rub
HCV: Hepatitis C virus
HR: Harm reduction
PWID: People who inject drugs
SSTI: Skin and soft tissue infections
WHO: World Health Organization
