# Supplementary material for: A two-component intervention to improve hand hygiene practices and promote alcohol-based hand rub use among people who inject drugs: a mixed-methods evaluation
Source: BMC Infect Dis. 2021 Feb 25;21:211. doi: 10.1186/s12879-021-05895-1 (PMC7905764; doi:10.1186/s12879-021-05895-1)
Supplement: Supplementary file 1 — Additional file 1: Supplementary Information. Questionnaires used during the study. [file 12879_2021_5895_MOESM1_ESM.docx]

**Supplementary Information. Questionnaires used during the study**

**Supplement to the article: A two-component intervention to improve hand hygiene practices and promote alcohol-based hand rub use among people who inject drugs: a mixed-methods evaluation**

Salim Mezaache^1,2^, Laélia Briand-Madrid^1,2^, Linda Rahni^1,2^, Julien Poireau^3^, Fiona Branchu^4^, Khafil Moudachirou^4,5^, Yourine Wendzinski^6^, Patrizia Carrieri^1,2^, Perrine Roux^1,2^

^1^Aix-Marseille Univ, INSERM, IRD, SESSTIM, Sciences économiques & sociales de la santé & traitement de l’information médicale, Marseille, France.

^2^ORS PACA, Observatoire régional de la santé Provence-Alpes-Côte d’Azur, Marseille, France.

^3^Nouvelle Aube, Marseille, France.

^4^Aides, Pantin, France.

^5^Laboratoire de recherche communautaire, Coalition Plus, Pantin, France.

^6^ASUD Nîmes, France.

**BASELINE QUESTIONNAIRE**

CODE: /___/___/ /___/___/

DATE : /___/___/ /___/___/ /___/___/

**Inclusion criteria**

**Q1. Age?** /__/__/__/

**Q2. During the past week, have you injected a substance?**

 0 No  1 Yes

**Q3. Do you speak French?**

 0 No  1 Yes
**If no, which language do you speak?** ______________________________*

**Q4. Are you staying in the city less than six weeks?**

 0 No  1 Yes

**Q5. To your knowledge, are you allergic to alcohol?**

 0 No  1 Yes

**1. Social, demographic and economic characteristics (DEM)**

**DEM1. Gender**

 0 Male

 1 Female

 2 Transgender

 98 DK

 99 NR

**DEM2. What is your school level?**

 0 Below high school degree

 1 Above or equal to high school degree

 98 DK

 99 NR

**DEM3. Are you living in a couple?**

 0 No ⏩ **go to question DEM4**

 1 Yes

 98 DK ⏩ **go to question DEM4**

 99 NR ⏩ **go to question DEM4**

**DEM3.1. If yes, is your partner a drug user?**

 0 No

 1 Yes, but do not inject

 2 Yes, with injection

 98 DK

 99 NR

**DEM4. Where are you living? (Only one choice possible)**

 0 In private housing

 1 In a therapeutic housing

 2 With your family

 3 In an hospital or a clinic

 4 In emergency housing

 5 With friends

 6 In an hotel

 7 In the street

 8 In a squat

 9 In a truck/trailer

 10 Other……………………………….

 98 DK

 99 NR

**DEM5. During the past month, did you spleep in the street?**

 0 No

 1 Yes

 98 DK

 99 NR

**DEM6. What is your employement situation ?**

 1 You are working (declared)

 2 You are working (undeclared)

 3 You are unemployed receiving public allowance

 4 You are student

 5 You are retired

 6 You are disable

 7 You are not employed and not receiving public allowance

 8 Other………………….

 98 DK

 99 NR

**2. Psychoactive substances consommations (CONS)**

**CONS1. How old were you when you injected drug for the first time?**

/___/___/ years old  98 DK  99 NR

**CONS2. Au cours du dernier mois, quelle a été votre consommation de substances psychoactives (y compris les médicaments prescrits)  ?**

| List of substances | **How many days per month?** | **How many times a day (average day)?** | **Prescribed?** | **Injected?** | **Main route**  1=oral  2=intraDKsal  3=smoked  4=injected |
| --- | --- | --- | --- | --- | --- |
| Heroin | /__/__/ | /__/__/ |  |  No  Yes | /__/ |
| Buprenorphine (1) | /__/__/ | /__/__/ |  No  Yes |  No  Yes | /__/ |
| Methadone | /__/__/ | /__/__/ |  No  Yes |  No  Yes | /__/ |
| Morphine (2) | /__/__/ | /__/__/ |  No  Yes |  No  Yes | /__/ |
| Other opioids (3) | /__/__/ | /__/__/ |  No  Yes |  No  Yes | /__/ |
| Cocaine | /__/__/ | /__/__/ |  |  No  Yes | /__/ |
| Crack or free base | /__/__/ | /__/__/ |  |  No  Yes | /__/ |
| Speedball | /__/__/ | /__/__/ |  |  No  Yes | /__/ |
| Amphetamines (4) | /__/__/ | /__/__/ |  |  No  Yes | /__/ |
| Methyphenidate (5) | /__/__/ | /__/__/ |  No  Yes |  No  Yes | /__/ |
| Benzodiazepines (6) | /__/__/ | /__/__/ |  No  Yes |  No  Yes | /__/ |
| Ketamine | /__/__/ | /__/__/ |  No  Yes |  No  Yes | /__/ |
| NPS or RC (7) | /__/__/ | /__/__/ |  |  No  Yes | /__/ |
| Psychedelics (8) | /__/__/ | /__/__/ |  |  No  Yes | /__/ |
| CanDKbis (9) | /__/__/ | /__/__/ |  |  |  |
| Other……………… | /__/__/ | /__/__/ |  No  Yes |  No  Yes | /__/ |

1. e.g. Subutex, Temgesic, Suboxone,…
2. e.g. SkeDKn®, Moscontin®, Lamaline®
3. e.g. péthidine, codéine, diDKcode, néocodion, efferalgan codéiné…
4. speed, MDMA, ecstasy
5. Ritaline, Concerta, Quasym
6. lexomil, lysanxia, noctran, rivotril, seresta, tranxene, valium, xaDKx
7. New Psychoactive Substances or Research Chemicals : synthetic canDKbinoïds, cathinones, 6-APB, 2C-B, MDPV, 4-MEX, méphédrone, PVP, 3-MMC, 4-MA, 4-FA, PPP, pipérazines, BZP, TFMPP, Methcat…
8. e.g. Artane, LSD/acide, champigNos, DMT, NBOMe…
9. weed, hasch, joints

**CONS3. With whom do you inject the most? (Only one choice possible)**

 0 Alone

 1 With your partner

 2 With a friend/family member

 3 In a group of people

 4 Other: ________________________

 98 DK

 99 NR

**CONS4. Where do inject more often? (Only one choice possible.)**

 0 In parking lots

 1 In public toilets

 2 In basements

 3 In strairwells

 4 In the street

 5 At home

 6 At someone’s house

 7 In a harm reduction centre

 8 In a drug consumption rool

 9 In parties

 10 Other: ____________________________________________________________

 98 DK

 99 NR

**CONS5. During the past month, how often have you injected drugs?**

 0 Less than once a week

 1 At least once a week

 2 Every days

 98 DK

 99 NR

**CONS5.1. How many times a day ?**

Average: /___/___/ injections per day

Maximum: /___/___/ injections per day

**3. Health problems (SANT)**

**SANT1 - During the past month, have you experienced one of these problems related to injecting drug use? (multiple choices possible)**

 0 No

 1 Cutaneous abcesses

 2 Other skin infection

 3 Necrosis

 4 Cotton fever

 5 Allergia

 6 Sepsis

 7 Endocarditis

 8 Other ………………………………….

 98 DK

 99 NR

**SANT 2 - During your life, have you experienced one of these problems related to injecting drug use? (multiple choices possible)**

 0 No

 1 Cutaneous abcesses

 2 Other skin infection

 3 Necrosis

 4 Cotton fever

 5 Allergia

 6 Sepsis

 7 Endocarditis

 8 Other ………………………………….

 98 DK

 99 NR

**4. Hand hygiene practices (HYG)**

**HYG1. During the past two weeks, how often did you wash your hands before injection?** 0 Never ⏩ **Go to question HYG2** 1 Less than half of the time
 2 Half of the time
 3 Most of the time
 4 Always
 98 DK ⏩ **Go to question HYG2** 99 NR ⏩ **Go to question HYG2**

If Yes:

**HYG.1.1 What did you use to wash your hands? (Multiple choices possible)**

 1 Only water

 2 Water + soap

 3 Alcohol-based handrubs

 4 Chlorexidine wipes

 5 Other…………………………

 98 DK

 99 NR

**HYG.1.1 What did you use, most often, to wash your hands? (Only one choice possible)**

 1 Only water

 2 Water + soap

 3 Alcohol-based handrubs

 4 Chlorexidine wipes

 5 Other…………………………

 98 DK

 99 NR

**WEEK 2 QUESTIONNAIRE**

CODE: /___/___/ /___/___/

DATE : /___/___/ /___/___/ /___/___/

**1. Hand hygiene practices (HYG)**

**HYG1. During the past two weeks, how often did you wash your hands before injection?** 0 Never ⏩ **Go to question HYG2** 1 Less than half of the time
 2 Half of the time
 3 Most of the time
 4 Always
 98 DK ⏩ **Go to question HYG2** 99 NR ⏩ **Go to question HYG2**

If Yes:

**HYG.1.1 What did you use to wash your hands? (Multiple choices possible)**

 1 Only water

 2 Water + soap

 3 Alcohol-based handrubs (other than MONORUB)

 4 MONORUB

 5 Chlorexidine wipes

 6 Other…………………………

 98 DK

 99 NR

**HYG.1.1 What did you use, most often, to wash your hands? (Only one choice possible)**

 1 Only water

 2 Water + soap

 3 Alcohol-based handrubs (other than MONORUB)

 4 MONORUB

 5 Chlorexidine wipes

 6 Other…………………………

 98 DK

 99 NR

**2. Technique of use of MONORUB (TECH)**

***We ask the participant to show his technique of use of MONORUB***

**TECH1. Which steps the participant have done properly?**

**TECH1.1 Step 1**

 0 No

 1 Yes

**TECH1.1 Step 2**

 0 No

 1 Yes

**TECH1.1 Step 3**

 0 No

 1 Yes

**TECH2. All the product have been used?**

 0 No

 1 Yes

**TECH4. Did the participant rub his hands during at least 15 seconds ?**

 0 No

 1 Yes

**3. Acceptability (ACC)**

***On a scale from 0 (not at all) to 10 (absolutely) :***

**ACC1. Overall, are you satisfied with the hand washing method? (packaging, product and technique)**

 0  1  2  3  4  5  6  7  8  9  10

**ACC2. Do you think this method was better than the one you were used to?**

 0  1  2  3  4  5  6  7  8  9  10

**ACC3. Do you think the packaging was easy to use?**

 0  1  2  3  4  5  6  7  8  9  10

**ACC4. Do you think the 3-step technique was easy?**

 0  1  2  3  4  5  6  7  8  9  10

**ACC5. Do you think you would show other people this method?**

 0  1  2  3  4  5  6  7  8  9  10

**ACC6. Pensez-vous que vous continueriez à utiliser ce produit s’il était disponible ?**

 0  1  2  3  4  5  6  7  8  9  10

**ACC7. Do you think you would continue to use this method if it were available free of charge?**

 0  1  2  3  4  5  6  7  8  9  10

**4. Safety of the product (SAFE)**

**SAFE1. Did you experience hand dryness?**

 0 No

 1 Yes

 98 DK
 99 NR

**SAFE2. Did you experience redness or burning on your hands?**

 0 No

 1 Yes

 98 DK
 99 NR

**SAFE3. Did you experience itching?**

 0 No

 1 Yes

 98 DK
 99 NR

**SAFE5. Did you experience other problems related to the product ?**

 0 No

 1 Yes…………………………

 98 DK
 99 NR

**SAFE6. If you have experienced one of those problems, did they prevent you from using the product?**

 0 No

 1 Yes

 98 DK
 99 NR

**SAFE7. Did you use the product for other than hand washing?**

 0 No

 1 Yes

 98 DK
 99 NR

**SAFE7.1. If Yes, which one? (Multiple choices possible)**

 1 Disinfection of injecting site

 2 Body hygiene

 3 Surface cleaning

 4 Combustible

 5 Ingestion

 6 Other

 98 DK

 99 NR

**WEEK 6 QUESTIONNAIRE**

CODE CONFIDENTIEL : /___/___/ /___/___/

DATE : /___/___/ /___/___/ /___/___/

LIEU : 0 Rue 1 Unité mobile 2 Salle de conso 3 CAARUD 4 Autre : ……………….

**1. Health problems (SANT)**

**SANT1 – Since the beginning of the study, have you experienced one of these problems related to injecting drug use? (multiple choices possible)**

 0 No

 1 Cutaneous abcesses

 2 Other skin infection

 3 Necrosis

 4 Cotton fever

 5 Allergia

 6 Sepsis

 7 Endocarditis

 8 Other ………………………………….

 98 DK

 99 NR

**1. Hand hygiene practices (HYG)**

**HYG1. During the past two weeks, how often did you wash your hands before injection?** 0 Never ⏩ **Go to question HYG2** 1 Less than half of the time
 2 Half of the time
 3 Most of the time
 4 Always
 98 DK ⏩ **Go to question HYG2** 99 NR ⏩ **Go to question HYG2**

If Yes:

**HYG.1.1 What did you use to wash your hands? (Multiple choices possible)**

 1 Only water

 2 Water + soap

 3 Alcohol-based handrubs (other than MONORUB)

 4 MONORUB

 5 Chlorexidine wipes

 6 Other…………………………

 98 DK

 99 NR

**HYG.1.1 What did you use, most often, to wash your hands? (Only one choice possible)**

 1 Only water

 2 Water + soap

 3 Alcohol-based handrubs (other than MONORUB)

 4 MONORUB

 5 Chlorexidine wipes

 6 Other…………………………

 98 DK

 99 NR

**2. Technique of use of MONORUB (TECH)**

***We ask the participant to show his technique of use of MONORUB***

**TECH1. Which steps the participant have done properly?**

**TECH1.1 Step 1**

 0 No

 1 Yes

**TECH1.1 Step 2**

 0 No

 1 Yes

**TECH1.1 Step 3**

 0 No

 1 Yes

**TECH2. All the product have been used?**

 0 No

 1 Yes

**TECH4. Did the participant rub his hands during at least 15 seconds ?**

 0 No

 1 Yes

**3. Acceptability (ACC)**

***On a scale from 0 (not at all) to 10 (absolutely) :***

**ACC1. Overall, are you satisfied with the hand washing method? (packaging, product and technique)**

 0  1  2  3  4  5  6  7  8  9  10

**ACC2. Do you think this method was better than the one you were used to?**

 0  1  2  3  4  5  6  7  8  9  10

**ACC3. Do you think the packaging was easy to use?**

 0  1  2  3  4  5  6  7  8  9  10

**ACC4. Do you think the 3-step technique was easy?**

 0  1  2  3  4  5  6  7  8  9  10

**ACC5. Do you think you would show other people this method?**

 0  1  2  3  4  5  6  7  8  9  10

**ACC6. Pensez-vous que vous continueriez à utiliser ce produit s’il était disponible ?**

 0  1  2  3  4  5  6  7  8  9  10

**ACC7. Do you think you would continue to use this method if it were available free of charge?**

 0  1  2  3  4  5  6  7  8  9  10

**4. Safety of the product (SAFE)**

**SAFE1. Did you experience hand dryness?**

 0 No

 1 Yes

 98 DK
 99 NR

**SAFE2. Did you experience redness or burning on your hands?**

 0 No

 1 Yes

 98 DK
 99 NR

**SAFE3. Did you experience itching?**

 0 No

 1 Yes

 98 DK
 99 NR

**SAFE5. Did you experience other problems related to the product ?**

 0 No

 1 Yes…………………………

 98 DK
 99 NR

**SAFE6. If you have experienced one of those problems, did they prevent you from using the product?**

 0 No

 1 Yes

 98 DK
 99 NR

**SAFE7. Did you use the product for other than hand washing?**

 0 No

 1 Yes

 98 DK
 99 NR

**SAFE7.1. If Yes, which one? (Multiple choices possible)**

 1 Disinfection of injecting site

 2 Body hygiene

 3 Surface cleaning

 4 Combustible

 5 Ingestion

 6 Other

 98 DK

 99 NR
